# County community health associations of net voting shift in the 2016 U.S. presidential election

**DOI:** 10.1371/journal.pone.0185051

**Published:** 2017-10-02

**Authors:** Jason H. Wasfy, Charles Stewart, Vijeta Bhambhani

**Affiliations:** 1 Cardiology Division, Massachusetts General Hospital, Harvard Medical School, Boston, Massachusetts, United States of America; 2 Department of Political Science, Massachusetts Institute of Technology, Cambridge, Massachusetts, United States of America; University of Minnesota, UNITED STATES

## Abstract

**Importance:**

In the U.S. presidential election of 2016, substantial shift in voting patterns occurred relative to previous elections. Although this shift has been associated with both education and race, the extent to which this shift was related to public health status is unclear.

**Objective:**

To determine the extent to which county community health was associated with changes in voting between the presidential elections of 2016 and 2012.

**Design:**

Ecological study with principal component analysis (PCA) using principal axis method to extract the components, then generalized linear regression.

**Setting:**

General community.

**Participants:**

All counties in the United States.

**Exposures:**

Physically unhealthy days, mentally unhealthy days, percent food insecure, teen birth rate, primary care physician visit rate, age-adjusted mortality rate, violent crime rate, average health care costs, percent diabetic, and percent overweight or obese.

**Main outcome:**

The percentage of Donald Trump votes in 2016 minus percentage of Mitt Romney votes in 2012 (“net voting shift”).

**Results:**

Complete public health data was available for 3,009 counties which were included in the analysis. The mean net voting shift was 5.4% (+/- 5.8%). Of these 3,009 counties, 2,641 (87.8%) had positive net voting shift (shifted towards Trump) and 368 counties (12.2%) had negative net voting shift (shifted away from Trump). The first principal component (“unhealthy score”) accounted for 68% of the total variance in the data. The unhealthy score included all health variables except primary care physician rate, violent crime rate, and health care costs. The mean unhealthy score for counties was 0.39 (SD 0.16). Higher normalized unhealthy score was associated with positive net voting shift (22.1% shift per unit unhealthy, p < 0.0001). This association was stronger in states that switched Electoral College votes from 2012 to 2016 than in other states (5.9% per unit unhealthy, p <0.0001).

**Conclusions and relevance:**

Substantial association exists between a shift toward voting for Donald Trump in 2016 relative to Mitt Romney in 2012 and measures of poor public health. Although these results do not demonstrate causality, these results suggest a possible role for health status in political choices.

## Background

The November 2016 U.S. presidential election featured major differences on health policy between the two major candidates, Hillary Clinton and Donald Trump.[1,2] In particular, the election was marked by broad disagreements about the future of the Affordable Care Act and occurred at a time of broad public disapproval of the U.S. health system.[3]

Also, the election was notable for substantial shifts in party voting, as compared with the 2012 presidential election.[4] Many analysts did not predict the result of the 2016 presidential election accurately, and longstanding patterns of voting in presidential elections shifted. In particular, in Texas, the Democratic candidate performed better than previous Democratic presidential nominees in any election in 20 years. However, in Wisconsin, the Republican nominee won for the first time in 32 years. Much is unknown about the sources of this shift in voting behavior. Education and race may have been associated with this shift.[5]

The role of health status of populations in this shift, however, is unclear. Previous work, mostly in political science, has generated some knowledge about the role of health in voting behavior. Specifically, both poor general health and poor mental health has been associated with reduced voter turnout[6–9] especially for older voters.[10] Fixed characteristics of individuals may explain part of the observation that voters who have voted previously are more likely to vote again.[11] Health appears to be one of those characteristics. Some evidence has suggested that voters with specific health conditions such as neurodegenerative diseases and substance use disorders are associated with very low turnout whereas voters sick with other conditions such as cancer and respiratory illnesses are associated with higher voter turnout.[10] This association between poor health and reduced voting turnout has been linked to more generous government health spending in areas with sicker voters.[12] Reduced voter turnout among sicker populations differs from their generally higher levels of other types of political engagement, such as direct advocacy with leaders and organization of demonstrations.[13] The known link between poor health and low turnout raises the possibility that health status of populations could be associated with net voting shifts.

In addition to the observed association between poor health and reduced turnout, some evidence suggests associations between health and voting for specific parties. In the United States, healthier voters have also been associated with voting for the Republican party.[7] Despite that, evidence in the literature also suggest that Republican voting is associated with some specific measures of poor health, such as obesity and low vaccination rates.[14,15] However, the overall association of party voting with health is unclear. Furthermore, little is known about associations between health and changes in voting behavior over time.

Understanding any association between health and changes in voting would provide insight to the role of social distress in influencing political change. Given the marked differences between the candidates’ positions on health policy and the possibility of health and other social factors as influential in the 2016 election results, we investigated associations between county community health and changes in party voting between the presidential elections of 2012 and 2016.

## Methods

### County-level study population

For 2016, presidential election data was downloaded from the Atlas of U.S. Presidential elections for all counties [16], except Alaska and states in New England. The number of votes for Alaska was obtained from the New York Times [17]. The number of votes for New England counties was obtained from official election returns released by state election departments. Presidential election data from 2012 was downloaded from The Guardian [18]. In 2016 and 2012, the number of counties available for analysis with voting data totaled 3,114 counties or county equivalents in the United States of America across all 50 states.

Public health measure data was obtained from the County Health Rankings and Roadmaps (CHR) Database through the Robert Wood Johnson Foundation program. Of 3,114 counties that had voting data, 3,009 (96.6%) had complete public health data. These 3,009 counties constituted the final sample size for analysis. All county level public health measures were merged with the 2016 and 2012 presidential data for analysis.

### Outcomes and covariates

The primary outcome was defined as percentage of Donald Trump votes in 2016 minus percentage of Mitt Romney votes in 2012 (“net voting shift”). In other words, the outcome was defined as the difference in percentage points between the two election cycles for the Republican candidate. This is defined mathematically in S7 Table.

Demographic predictor variables used for controls to analyze whether public health status was associated with the difference in Republican nominee votes between 2016 and 2012 included percent female (gender), percent 65 and over (age), percent that have attended some level of college (education), percent rural (environment), population estimates, percent without English proficiency, percent non-Hispanic white (white), percent Hispanic (Hispanic), percent African American (African American), household income (social class), and health care costs. These demographic predictors were obtained through the CHR website. All demographic predictors were derived from the U.S. Census Population Estimates from 2014, except percent rural, which was from the U.S. Census in 2010, and percent without English proficiency which was from the American Community Survey (2010–2014). Health care costs, also available through the CHR website, were aggregated from Dartmouth Atlas calculations from Medicare claims files. As noted on the Dartmouth Atlas website, these patient-level average health care costs include Medicare Part A and Part B claims for patients in fee-for-service Medicare, after adjustment for age, gender, and race as well as regional differences in prices.[19]

Variables from the CHR database were chosen that represented measures of community health plausibly related to voting behavior. Variables that had high proportions of missing values (including rates of drug-overdose deaths) were eliminated. The 10 public health measures used to create a scale variable to define unhealthy public health status were (1) physically unhealthy days, defined as the age-adjusted mean number of physically unhealthy days reported in the past 30 days (2) mentally unhealthy days, defined as the age-adjusted mean number of mentally unhealthy days reported in the past 30 days (age-adjusted) (3) percent food insecure (4) teen birth rate per 1,000 female population, ages 15–19 (5) primary care physician visit rate per 100,000 people per year (6) age-adjusted mortality rate per 100,000 people per year (7) violent crime rate, defined as the number of reported violent crime offenses per 100,000 people per year (8) average health care costs per person (9) percent diabetic and (10) percent overweight or obese, defined as percentage of adults that report a body mass index of 30 or more.

### Statistical analysis

Continuous predictor variables are reported as mean ± standard deviation (SD). Principal component analysis (PCA) was used to reduce predictor public health variables as the public health measures were highly correlated. In this analysis, all ten public health predictors were entered into the PCA and any variables that loaded on more than one component was removed from the PCA. PCA is often used when there is a large sample size (>100 observations) and a reduction of multicollinearity is needed.^10^ The public health predictor variables were subjected to PCA using principal axis method to extract the components. This was followed by a varimax (orthogonal rotation) of the data. This converted the dataset from correlated variables into a set of values of linearly uncorrelated variables (principal components). A principal component is defined as a function of the variables and is the weighted linear sum of variables in analysis that accounts for a large amount of variation in the data^10^. For the PCA, we used eigenvalues > 1 to determine the number of factor components to retain. We also used a scree test to graphically display the size of the eigenvalue associated with each component.^10^ Standardized scoring coefficients were then assigned to each variable for each component that was retained. This process removed 3 variables, primary care physician rate, violent crime rate, and health care costs (also see Results section). Therefore, the retained variables were: (1) physically unhealthy days (2) mentally unhealthy days, (3) percent food insecure (4) teen birth rate (5) age-adjusted mortality rate per 100,000 people per year (6) percent diabetic and (7) percent overweight or obese.

Once the PCA was complete, we reduced the remaining public health variables that were not removed into one public health status measure from the first principal component (FPC) and defined it as the “unhealthy” component. The “unhealthy” component was then normalized to a [0, 1] scale. Each county was then assigned an “unhealthy” score. This “unhealthy” score was then used as a predictor variable in a generalized linear regression model.

We then fit a generalized linear regression model with the unhealthy variable as the predictor for the primary outcome variable, net voting shift. We also evaluated the association between the “unhealthy” status and our outcome after adjusting for percent female (gender), percent 65 and over (age), percent that have attended some level of college (education), percent rural (environment), total population, percent without English proficiency, percent non-Hispanic White (race), percent Hispanic (race), percent African American (race), household income (social class), and health care costs. We transformed the population to log(population) because the data was right-skewed. Including population in the model has the effect of addressing potential omitted variables bias, given an association between more populous counties and Democratic party voting. In both models, unadjusted and adjusted, we included state-level fixed effects in order to absorb out the effect of the state variable and account for clustering effects in specific states. For example, campaign focus on a specific state might be expected to affect net voting shift in all counties within that state. We also applied an analytical weight for the total number of votes in 2016 in each county. This is standard practice in ecological election analysis, and the coefficient of this weight estimates the coefficient that would have been obtained if it were possible to perform the same regression with individual-level data.[20] Results from the generalized linear regression model are presented as 1-unit increase in predictor variable with p values. P values < 0.05 were considered significant.

### Sensitivity analyses

To explore potential effect modification of region on the relationship between the unhealthy score and net voting shift, as a sensitivity analysis, we repeated the main regression analysis with interaction terms representing the regions of the country (Midwest, Northeast, South, and West). In addition, to further explore the implications of our main findings, we conducted an additional analysis including an interaction term of states in which Electoral College delegates switched from the Democratic party in 2012 to the Republican Party in 2016 (“swing state analysis”). These states include Iowa, Pennsylvania, Wisconsin, Ohio, Michigan, and Florida. Since the 2^nd^ Congressional District of Maine allocates Electoral College delegates separately, we also included counties in the 2^nd^ Congressional District of Maine. Kennebec County, the only county in Maine that is in both congressional districts, was considered to be in the 1^st^ Congressional District for the purposes of this analysis given that the population within the county is greater within the 1^st^ Congressional District. Finally, to explore potential confounding between county community health and county-level change in aggregate turnout, we performed a separate analysis with change in voting turnout as the dependent variable and the unhealthy score and demographic covariates as independent variables. In this separate analysis, county-level change in turnout was defined as difference change in total vote between 2016 and 2012 divided by county population.

In addition, to further explore the association of percent rural and percent white with net voting shift, we conducted auxiliary regressions and re-ran the main model with different combinations of explanatory variables. Detailed methods and results from these analyses appear in the S5 Table and S6 Table.

Analyses were done with the use of SAS version 9.4 software. This analysis was exempt from review by the Institutional Review Board at Partners Healthcare, since no patient level data was used.

## Results

Complete public health data was available for 3,009 counties which were included in the analysis. The healthiest counties were Pitkin County, Colorado (unhealthy score 0.02579), Loudoun County, Virginia (unhealthy score 0.02581), Carver County, Minnesota (unhealthy score 0.02771). The unhealthiest counties were Oglala Lakota County, South Dakota (unhealthy score 1.00000), Wilcox County, Alabama (unhealthy score 0.95606), and Holmes County, Mississippi (unhealthy score 0.95252).

The mean county net voting shift was 5.4% (+/- 5.8%). Of these 3,009 counties, 2,641 (87.8%) had positive net voting shift (shifted towards Trump) and 368 counties (12.2%) had negative net voting shift (shifted away from Trump). Counties with positive net voting shift had higher proportions of white population (79% vs. 65%, p <0.0001), higher proportion of rural population (63% vs. 21%, p = <0.0001), and lower average household income ($45,142 vs. $60,086, p <0.0001). Counties with positive net voting shift also had higher teenage birth rates (43 vs. 31 births per 1,000 female population, p < 0.0001), higher age-adjusted mortalities (401 vs. 301 deaths per 100,000 population, p < .0001), but lower rates of violent crime (239 vs. 312 offenses per 100,000 population, p < .0001). Characteristics of counties with positive and negative net voting shifts are shown in Table 1.

**Table 1:** Baseline demographics of county-level data*.

Variable	Overall	Net voting shift towards Trump	Net voting shift away from Trump	P
	n = 3,009	n = 2641	n = 368	
**Demographics**				
% Female	50 (2)	50 (2)	51 (1)	< .0001
% 65 and over	17 (4)	18 (4)	14 (4)	< .0001
% Some College	56 (11)	54 (11)	67 (11)	< .0001
% Rural	57 (31)	63 (29)	21 (24)	< .0001
% Not proficient in English	2 (3)	1 (3)	4 (4)	< .0001
% Non-Hispanic White	77 (20)	79 (19)	65 (21)	< .0001
% Hispanic	9 (14)	8 (12)	18 (18)	< .0001
% African American	9 (14)	9 (15)	10 (13)	0.2077
Household Income	$46,970 ($12,141)	$45,142 ($10,079)	$60,086 ($16,762)	< .0001
Population	1,056,699 (332,630)	58,956 (140, 498)	440,905 (797,785)	< .0001
**Public Health Measures**				
Physically Unhealthy Days	4 (1)	4 (1)	3 (1)	< .0001
Mentally Unhealthy Days	4 (1)	4 (1)	3 (0.5)	< .0001
% Food Insecure	15 (4)	15 (4)	14 (4)	< .0001
Teen Birth Rate	42 (19)	43 (19)	31 (18)	< .0001
PCP Rate	56 (33)	52 (30)	86 (41)	< .0001
Age-Adjusted Mortality	390 (102)	402 (98)	301 (81)	< .0001
Violent Crime Rate	252 (196)	243 (190)	315 (227)	< .0001
Health care costs	$9,377 ($1,433)	$9,444 ($1,435)	$8,889 ($1,321)	< .0001
% Diabetic	11 (2)	11 (2)	9 (2)	< .0001
% Obese	31 (4)	32 (4)	26 (5)	< .0001
**Outcome**				
% Donald Trump—% Mitt Romney	5.8 (5.4)	7.1 (4.3)	-3.4 (3.0)	< .0001

*continuous variables reported as mean +/- standard deviation

### Principal component analyses

When the 10 public health predictor variables initially were subjected to a PCA, after removing the 3 variables that had a meaningful loading on more than one component (primary care physician rate, violent crime rate, and health care costs), the PCA was run again with only the remaining variables. Therefore, only 7 public health variables were included. This was followed by a varimax (orthogonal rotation) of the data. Only the first principal component (FPC) displayed eigenvalue greater than 1, and the results of a scree test also suggested that only the FPC was meaningful. Therefore, only the FPC was retained for rotation. The FPC accounted for 68% of the total variance in the data. The FPC, also known as the “unhealthy” component, was the weighted linear sum of variables: physically unhealthy days, mentally unhealthy days, percent of food insecurity, teen birth rate, age-adjusted mortality, percent diabetic, and percent obese. Furthermore, physically unhealthy days and age-adjusted mortality had the highest standardized scoring coefficients for the “unhealthy” component.

Public health measures, eigenvalues, corresponding factor loadings, and standardized scoring coefficients for the unhealthy component are presented in S1, S2 and S3 Tables. The resulting definition of the “unhealthy” variable also appears in S3 Table.

### Regression analysis

The mean unhealthy score for counties was 0.39 (SD 0.16) on a normalized scale. Higher normalized unhealthy score was associated with positive net voting shift in an unadjusted model (22.1% shift per unit unhealthy, p < 0.0001). When adjusted for demographic variables in a generalized linear model, each unhealthy point was associated with net voting shift of 4.1% (p = 0.0068). All demographic variables except % rural (p = 0.1249) and % non-Hispanic white (p = 0.0890) remained significantly associated with the net voting shift. Full results of the model appear in Table 2.

**Table 2:** Generalized linear regression estimates adjusted for demographic variables and unhealthy component.

Parameter	% Net voting shift	Standard Error	P
Unhealthy	4.1048	1.5143	0.0068
% Female	-0.0958	0.0574	0.0950
% 65 and over	0.0688	0.0198	0.0005
% Some College Education	-0.1374	0.0119	< .0001
% Rural	0.0070	0.0046	0.1249
Log Population	-0.9520	0.0849	< .0001
% Not proficient in English	-0.2654	0.0392	< .0001
% Non-Hispanic white	-0.0246	0.0144	0.0890
% Hispanic	-0.0699	0.0132	< .0001
% African American	-0.1095	0.0148	< .0001
Household Income	-0.0001	0.0000	< .0001
Healthcare costs	0.0009	0.0001	< .0001

### Region-level and swing state sensitivity analyses

In a model that assessed the independent effect of region on the relationship between the unhealthy score and net voting shift after adjustment for demographics, there was no effect modification of the Northeast region on the relationship between the unhealthy score and net voting shift, relative to the Midwest (p = 0.46). The net voting shift in the South per unit unhealthy score was 9.8% less and in the West was 8.6% less, both relative to the Midwest (p < 0.0001 for both). Full results of the sensitivity analysis with regional interaction terms appear in Table 3.

**Table 3:** Interaction of unhealthy factor with region.

Region	% Voting Shift*	Standard Error	P
Midwest (Reference)	ref	—	—
Northeast	-1.26	1.70	0.46
South	-9.83	1.35	<0.0001
West	-8.62	1.75	<0.0001

*adjusted for demographic variables

In a model that included an interaction term between the unhealthy score and states that switched Electoral College delegates in the 2016 election relative to the 2012 election (including the 2^nd^ Congressional District of Maine), the interaction between the unhealthy score and swing state status was significant (5.9% per unit unhealthy compared with non-swing states, p <0.0001). Results are in S4 Table.

After adjustment for demographic variables and absorbing out state-level effects, there was no county-level association in a model between the unhealthy score and aggregate voting turnout (p = 0.836).

## Discussion

We have shown here that poor public health is associated with an aggregate shift towards voting Republican in 2016 compared with 2012. In particular, there was a statistically significant association between this voting trend and nearly every examined measure of public health. These results are important for several reasons. This election was marked by substantial relative differences in party voting. In some cases, states that had traditionally supported Republican presidential candidates shifted away from the Republican nominee while states that had traditionally supported democratic presidential candidates shifted towards the Republican nominee. These shifts realigned patterns of party voting. Our results here suggest that aggregate health status was associated with these shifts in voting behavior between 2012 and 2016.

We have also demonstrated that this association of net voting shift with health status was stronger in states that changed political parties from 2012 to 2016. The association of net voting shift per unit of the unhealthy score was 5.9% greater in Iowa, Pennsylvania, Wisconsin, Michigan, Ohio, Florida, and the 2^nd^ Congressional District of Maine than in states that did not shift Electoral College delegates from 2012 to 2016. In that context, although our results cannot demonstrate causality, our results suggest a possible role of public health in determining the ultimate outcome of the overall election.

Furthermore, our results extend previous findings on party affiliation and public health to include a change in party voting.[21–23] Especially given that the 2016 election was interpreted by many as an expression of dissatisfaction with the status quo, our results suggest that poor public health was associated with social and political change. Although previous analyses have demonstrated an association between Republican voting and better health[7], our results here demonstrate that a net voting shift towards a Republican presidential candidate was associated with poor health. Several possibilities could explain that discrepancy. One possibility is that relatively sicker voters did not vote at all in 2012 and voted for the Republican candidate in 2016. Alternatively, a broader political realignment may be shifting sicker populations to Republican voting patterns, compared to what had been observed previously. In that sense, our results are compatible with recent findings that changes in county life expectancy between 1980 and 2016 were correlated with changes in party presidential voting between 2008 and 2016[24] although our analysis here focuses on a shorter time interval. The health variables here are likely correlated with observed changes in life expectancy reported previously.

However, it is also possible that sicker individuals within sicker communities did not actually shift their votes. It is critical to interpret our results as a county-based ecological association rather than an analysis of individual voting behavior. We cannot know from these data how individuals voted. Sicker individuals may not have shifted their votes. In fact, sicker individual voters may have shifted their preferences to the Democratic candidate but also had lower turnout, perhaps because of their illnesses.

Alternatively, unmeasured social or cultural confounders of counties with sicker individuals may have predisposed voting towards the Republican candidate in 2016, more than in 2012. As such, the shift of counties may be attributable to healthier voters within those counties. The sicker individual voters themselves may not have shifted voting at all, or may have even shifted towards the Democratic candidate in 2016. Previous work has demonstrated that in poorer, more Republican states, poorer voters tend strongly towards Democratic voting (even more strongly than in Democratic states).[25] Our results here may suggest that this type of effect could exist for health as well as income. Comparisons of voting behavior among sicker individuals and sicker communities could explore this possibility further.

Finally, since President Trump has advocated for the repeal of the Affordable Care Act, which has been projected to increase uninsurance by 24 million Americans[26], sicker populations shifting votes to the Republican candidate may represent voters voting against their self-interest. Addressing that possibility with these data is complex for several reasons. Ultimately, whether policies promoted by the winning candidate in 2016 will improve or worsen community health is not specifically addressable from these data. These types of analyses would need to assess changes in health status over time, controlling for preexisting trends. Furthermore, as an analysis of community health within counties, we cannot analyze the voting behavior of the specific voters within counties, for example, who would lose health insurance because of potential repeal of the Affordable Care Act.

Our study should be interpreted in the setting of important limitations. First, although we have demonstrated an association between public health and voting behavior we cannot exclude that underlying unmeasured social factors confounded these results. In particular, from this analysis, we cannot make causal claims to link community health and voting. For example, social distress or other factors may have affected both net voting shift and community health. Second, as a county-level analysis we cannot examine individual predictors of voting behavior. Finally, our measures of public health did not include effects of the opioid epidemic, which may also have an association with both other measures of public health and voting behavior and as such may introduce confounding into this analysis.

In conclusion, we have demonstrated an association between poor public health and a shift toward voting for the Republican candidate in 2016 compared with 2012. These results demonstrate an association between poor public health and political choices in an election that produced dramatic change. This association was particularly strong in states that switched political parties in the Electoral College. In that context, these results raise the possibility that poor public health was associated with voting and the outcome of the election.

## Supporting information

S1 File Final election minimal data set. Dataset used for main analysis.
(SAS7BDAT)

S1 Table Eigenvalues and proportion of variance explained. Variance attributable to each eigenvalue.
(DOCX)

S2 Table Principal component analysis. Correlation between public health variables and the unhealthy component.
(DOCX)

S3 Table Standardized scoring coefficients of unhealthy component. Standardized coefficients of correlation between public health variables and the unhealthy component.
(DOCX)

S4 Table Interaction of unhealthy factor with states that switched from 2012 to 2016. Interaction analysis of swing states.
(DOCX)

S5 Table Full model without hispanic and african american variables. Sensitivity analysis to explore relationship between race and voting shift.
(DOCX)

S6 Table Full model without hispanic, African American, and population variables. Sensitivity analysis to explore relationship between race, county population, and voting shift.
(DOCX)

S7 Table Definitions of voting variables. Definitions of net voting shift and difference in voting turnout.
(DOCX)
