# Supplementary material for: County community health associations of net voting shift in the 2016 U.S. presidential election
Source: PLoS One. 2017 Oct 2;12(10):e0185051. doi: 10.1371/journal.pone.0185051 (PMC5624580; doi:10.1371/journal.pone.0185051)
Supplement: S1 Table — Variance attributable to each eigenvalue. (DOCX) [file pone.0185051.s002.docx]

After the principal components analysis, we grouped the 7 variables – physically unhealthy days, mentally unhealthy days, % food insecure, teen birth rate, age-adjusted mortality, percent diabetic, and percent obese into one variable termed “unhealthy”. We then standardized the scores for each variable.

| **Supplemental Table S1. Eigenvalues and Proportion of Variance Explained** | | | | |
| --- | --- | --- | --- | --- |
| **Component** | **Eigenvalue** | **Difference** | **Proportion of Variance Explained** | **Cumulative Variance Explained** |
| 1 | 4.731 | 4.021 | 0.676 | 0.676 |
| 2 | 0.710 | 0.157 | 0.101 | 0.777 |
| 3 | 0.553 | 0.150 | 0.079 | 0.856 |
| 4 | 0.403 | 0.067 | 0.058 | 0.914 |
| 5 | 0.335 | 0.124 | 0.048 | 0.962 |
| 6 | 0.211 | 0.153 | 0.030 | 0.992 |
| 7 | 0.058 |  | 0.008 | 1.000 |
| *Component 1 was renamed “Unhealthy" Component | |  |  |  |
