# Supplementary material for: County community health associations of net voting shift in the 2016 U.S. presidential election
Source: PLoS One. 2017 Oct 2;12(10):e0185051. doi: 10.1371/journal.pone.0185051 (PMC5624580; doi:10.1371/journal.pone.0185051)
Supplement: S2 Table — Correlation between public health variables and the unhealthy component. (DOCX) [file pone.0185051.s003.docx]

| **Supplemental Table S2. Principal Component Analysis** | |
| --- | --- |
| **Public Health Variables** | **Unhealthy Component** |
| Physically Unhealthy Days | 0.91451 |
| Mentally Unhealthy Days | 0.83648 |
| % Food Insecure | 0.8023 |
| Teen Birth Rate | 0.77438 |
| Age-Adjusted Mortality | 0.87399 |
| % Diabetic | 0.85549 |
| % Obese | 0.67485 |
