# Supplementary material for: County community health associations of net voting shift in the 2016 U.S. presidential election
Source: PLoS One. 2017 Oct 2;12(10):e0185051. doi: 10.1371/journal.pone.0185051 (PMC5624580; doi:10.1371/journal.pone.0185051)
Supplement: S3 Table — Standardized coefficients of correlation between public health variables and the unhealthy component. (DOCX) [file pone.0185051.s004.docx]

| **Supplemental Table S3. Standardized Scoring Coefficients of Unhealthy Component** | |
| --- | --- |
| **Public Health Variables** | **Unhealthy Component** |
| Physically Unhealthy Days | 0.19332 |
| Mentally Unhealthy Days | 0.17683 |
| % Food Insecure | 0.1696 |
| Teen Birth Rate | 0.1637 |
| Age-Adjusted Mortality | 0.18475 |
| % Diabetic | 0.18084 |
| % Obese | 0.14266 |

The following formula for predicting “unhealthy” is as follows:

Y = 0.19332 * Physically Unhealthy Days + 0.17683 * Mentally Unhealthy Days + 0.16960 * % Food Insecure + 0.16370*Teen Birth Rate + 0.18475*Age-Adjusted Mortality + 0.18084* %Diabetic + 0.14266*%Obese
