# Supplementary material for: County community health associations of net voting shift in the 2016 U.S. presidential election
Source: PLoS One. 2017 Oct 2;12(10):e0185051. doi: 10.1371/journal.pone.0185051 (PMC5624580; doi:10.1371/journal.pone.0185051)
Supplement: S4 Table — Interaction analysis of swing states. (DOCX) [file pone.0185051.s005.docx]

| **Supplemental Table S4. Interaction of Unhealthy Factor with States that switched from 2012 to 2016** | | | |
| --- | --- | --- | --- |
| **Switch** | **% Voting Shift** | **Standard Error** | **P** |
| States that did not switch | ref | -- | -- |
| States that did switch | 5.91 | 1.31 | <.0001 |

*adjusted for demographic variables

*States that switched included: Iowa, Pennsylvania, Wisconsin, Michigan, Ohio, and Florida and certain counties of Maine’s 2^nd^ congressional district that included: Androscoggin, Aroostook, Franklin, Hancock, Oxford, Penobscot, Piscataquis, Somerset, Waldo, Washington
