# Supplementary material for: County community health associations of net voting shift in the 2016 U.S. presidential election
Source: PLoS One. 2017 Oct 2;12(10):e0185051. doi: 10.1371/journal.pone.0185051 (PMC5624580; doi:10.1371/journal.pone.0185051)
Supplement: S5 Table — Sensitivity analysis to explore relationship between race and voting shift. (DOCX) [file pone.0185051.s006.docx]

Additional analyses exploring association of net voting shift with race and rural/urban

To further explore associations between the variables used to adjust in the full model, we first conducted auxiliary regressions with “white” and “rural” respectively as the dependent variable and the other dependent variables still as dependent variables. We then re-ran the main model, with variables associated with these other independent variables omitted.

For “white,” in an auxiliary regression, we found that (as expected) there is an inverse association between “white” and “Hispanic” (-0.927, p < 0.0001) as well as “white” and “African American” (-0.824, p < 0.0001). These findings are consistent with the expected conclusion that even after adjustment for the other variables, counties that have higher percentages of white population have lower percentages of African American and Hispanic population.

Therefore we then re-ran the full model with “African American” and “Hispanic” removed. In this model, the association between “white” and net voting shift became significant (0.0665 points per percentage white population, p <0.0001):

**Supplemental Table S5. Full Model Without Hispanic and African American Variables**

| **Parameter** | **Estimate** | **Standard Error** | **P** |
| --- | --- | --- | --- |
| **unhealthy** | 5.06951 | 1.47181 | 0.0006 |
| **% Female** | -0.1348 | 0.05763 | 0.0194 |
| **% 65 and over** | 0.06968 | 0.0199 | 0.0005 |
| **% Some College** | -0.1232 | 0.01137 | <.0001 |
| **% Rural** | 0.01179 | 0.00452 | 0.0092 |
| **Log population** | -0.9245 | 0.08443 | <.0001 |
| **% Not Proficient in English** | -0.1666 | 0.03202 | <.0001 |
| **% Non Hispanic White** | 0.06654 | 0.00754 | <.0001 |
| **Household Income** | -6E-05 | 8.1E-06 | <.0001 |
| **Health care costs** | 0.00081 | 6.8E-05 | <.0001 |
