# Supplementary material for: County community health associations of net voting shift in the 2016 U.S. presidential election
Source: PLoS One. 2017 Oct 2;12(10):e0185051. doi: 10.1371/journal.pone.0185051 (PMC5624580; doi:10.1371/journal.pone.0185051)
Supplement: S6 Table — Sensitivity analysis to explore relationship between race, county population, and voting shift. (DOCX) [file pone.0185051.s007.docx]

For “rural,” in an auxiliary regression, we found that there is an inverse association between the population of the county and “rural” (-13.37, p <0.0001). These findings are consistent with the conclusion that even after adjustment for the other variables, rural counties are associated with lower populations.

Therefore we re-ran the full model with county population removed (as well as without African American and Hispanic). In this model, “rural” became significant (0.0415, p < 0.0001).

**Supplemental Table S6. Full Model Without Hispanic, African American, and Population Variables**

| **Parameter** | **Estimate** | **Standard Error** | **P** |
| --- | --- | --- | --- |
| **unhealthy** | 9.27801492 | 1.44912 | <.0001 |
| **% Female** | -0.23695656 | 0.058 | <.0001 |
| **% 65 and over** | 0.08639087 | 0.02023 | <.0001 |
| **% Some College** | -0.12218034 | 0.0116 | <.0001 |
| **% Rural** | 0.04149267 | 0.00369 | <.0001 |
| **% Not Proficient in English** | -0.15725103 | 0.03265 | <.0001 |
| **% Non Hispanic White** | 0.08823996 | 0.00742 | <.0001 |
| **Household Income** | -4.3082E-05 | 8.2E-06 | <.0001 |
| **Health care costs** | 0.00053799 | 6.4E-05 | <.0001 |

We believe that these additional analyses demonstrate association between counties with higher proportions of white voters and rural counties with positive net voting shift (towards Trump). We also believe that these results suggest that after adjustment for the other variables in the full model, there is not statistically significant association between white and rural and net voting shift but lack of association is attributable to other related variables in the model.

In all of these additional models, there exists association between the unhealthy variable and positive net voting shift (toward Trump), supporting the robustness of our main findings.
