# Supplementary material for: County community health associations of net voting shift in the 2016 U.S. presidential election
Source: PLoS One. 2017 Oct 2;12(10):e0185051. doi: 10.1371/journal.pone.0185051 (PMC5624580; doi:10.1371/journal.pone.0185051)
Supplement: S7 Table — Definitions of net voting shift and difference in voting turnout. (DOCX) [file pone.0185051.s008.docx]

**Supplemental Table S7. Definitions of Voting Variables**

Definition of Net voting shift

Net voting shift = % Donald Trump 2016 - % Mitt Romney 2012

The percentage of Donald Trump votes for 2016 election cycle was calculated as such:

% Donald Trump = # Donald Trump Votes / (# Donald Trump votes + # Hillary Clinton votes)

The percentage of Mitt Romney votes for 2012 election cycle was calculated as such:

% Mitt Romney = # Mitt Romney Votes / (# Mitt Romney votes + # Barack Obama votes)

All other candidates were excluded from the denominator.

Definition of Difference in Voter Turnout

% Difference in Voter Turnout = [(Total 2016 votes – Total 2012 votes) / County Population]*100
